# Supplementary material for: The Effect of Nattokinase-Monascus Supplements on Dyslipidemia: A Four-Month Randomized, Double-Blind, Placebo-Controlled Clinical Trial
Source: Nutrients. 2023 Sep 30;15(19):4239. doi: 10.3390/nu15194239 (PMC10574353; doi:10.3390/nu15194239)
Supplement: Supplementary file 1 [file nutrients-15-04239-s001.zip › nutrients-2581191-supplementary.pdf]

Supplements

We have a power of 93.91% at a 0.05 two-sided significance level to detect the absolute change from baseline to 120 days for TC (Supplement Table S1).

Supplement Table S1 Power analysis of primary and secondary outcomes in the intention-to-treat population

Variables	power
Primary outcomes	
TC	
Baseline, mmol/L	0.1204
45-days, mmol/L	0.9100
Absolute change from baseline to 45-days, mmol/L	0.9578
Percentage change from baseline to 45-days, %	0.9817
120-days, mmol/L	0.7679
Absolute change from baseline to 120-days, mmol/L	0.9391
Percentage change from baseline to 120-days, %	0.9786
TG	
Baseline, mmol/L	0.0678
45-days, mmol/L	0.2846
Absolute change from baseline to 45-days, mmol/L	0.1796
Percentage change from baseline to 45-days, %	0.2277
120-days, mmol/L	0.4616
Absolute change from baseline to 120-days, mmol/L	0.2796
Percentage change from baseline to 120-days, %	0.2218
LDL-C	
Baseline, mmol/L	0.1339
45-days, mmol/L	0.9461
Absolute change from baseline to 45-days, mmol/L	0.9983
Percentage change from baseline to 45-days, %	0.9992
120-days, mmol/L	0.7239
Absolute change from baseline to 120-days, mmol/L	0.9896
Percentage change from baseline to 120-days, %	0.9946
non-HDL-C	
Baseline, mmol/L	0.0788
45-days, mmol/L	0.9684
Absolute change from baseline to 45-days, mmol/L	0.9836
Percentage change from baseline to 45-days, %	0.9947
120-days, mmol/L	0.8713
Absolute change from baseline to 120-days, mmol/L	0.9400
Percentage change from baseline to 120-days, %	0.9843
LDL-C to HDL-C ratio	

Variables	power
Baseline	0.0541
45-days	0.8550
Absolute change from baseline to 45-days	1.0000
Percentage change from baseline to 45-days, %	1.0000
120-days	0.6128
Absolute change from baseline to 120-days	0.9773
Percentage change from baseline to 120-days, %	0.9899
HDL-C	
Baseline, mmol/L	0.1575
45-days, mmol/L	0.3106
Absolute change from baseline to 45-days, mmol/L	0.1656
Percentage change from baseline to 45-days, %	0.1327
120-days, mmol/L	0.1341
Absolute change from baseline to 120-days, mmol/L	0.0516
Percentage change from baseline to 120-days, %	0.0517
Second outcomes	
Right CIMT	
Baseline, mm	0.0849
120-days, mm	0.0627
Absolute change from baseline to 120-days, mm	0.1674
Percentage change from baseline to 120-days, %	0.0667
Left CIMT	
Baseline, mm	0.0514
120-days, mm	0.0516
Absolute change from baseline to 120-days, mm	0.0617
Percentage change from baseline to 120-days, %	0.0507
Average (Left and right) CIMT	
Baseline, mm	0.0570
120-days, mm	0.0537
Absolute change from baseline to 120-days, mm	0.0898
Percentage change from baseline to 120-days, %	0.0829

Supplement Table S2 Consort 2010 checklist

CONSORT 2010 checklist of information to include when reporting a randomised trial*

Section/Topic	Item No	Checklist item	Reported on page No
Title and abstract			
	1a	Identification as a randomised trial in the title	1

	1b	Structured summary of trial design, methods, results, and conclusions (for specific guidance see CONSORT for abstracts)	1
Introduction			
Background and objectives	2a	Scientific background and explanation of rationale	1-2
	2b	Specific objectives or hypotheses	2
Methods			
Trial design	3a	Description of trial design (such as parallel, factorial) including allocation ratio	2
	3b	Important changes to methods after trial commencement (such as eligibility criteria), with reasons	NA
Participants	4a	Eligibility criteria for participants	2
	4b	Settings and locations where the data were collected	2
Interventions	5	The interventions for each group with sufficient details to allow replication, including how and when they were actually administered	3
Outcomes	6a	Completely defined pre-specified primary and secondary outcome measures, including how and when they were assessed	4
	6b	Any changes to trial outcomes after the trial commenced, with reasons	NA
Sample size	7a	How sample size was determined	2
	7b	When applicable, explanation of any interim analyses and stopping guidelines	2-3
Randomisation:			
Sequence generation	8a	Method used to generate the random allocation sequence	2-3
	8b	Type of randomisation; details of any restriction (such as blocking and block size)	2-3
Allocation concealment mechanism	9	Mechanism used to implement the random allocation sequence (such as sequentially numbered containers), describing any steps taken to conceal the sequence until interventions were assigned	2-3
Implementation	10	Who generated the random allocation sequence, who enrolled participants, and who assigned participants to interventions	2-3
Blinding	11a	If done, who was blinded after assignment to interventions (for example, participants, care providers, those assessing outcomes) and how	3
	11b	If relevant, description of the similarity of interventions	3

Statistical methods	12a	Statistical methods used to compare groups for primary and secondary outcomes	4
	12b	Methods for additional analyses, such as subgroup analyses and adjusted analyses	4
Results			
Participant flow (a diagram is strongly recommended)	13a	For each group, the numbers of participants who were randomly assigned, received intended treatment, and were analysed for the primary outcome	3
	13b	For each group, losses and exclusions after randomisation, together with reasons	3
Recruitment	14a	Dates defining the periods of recruitment and follow-up	2-3
	14b	Why the trial ended or was stopped	3
Baseline data	15	A table showing baseline demographic and clinical characteristics for each group	5
Numbers analysed	16	For each group, number of participants (denominator) included in each analysis and whether the analysis was by original assigned groups	3
Outcomes and estimation	17a	For each primary and secondary outcome, results for each group, and the estimated effect size and its precision (such as 95% confidence interval)	6-8
	17b	For binary outcomes, presentation of both absolute and relative effect sizes is recommended	NA
Ancillary analyses	18	Results of any other analyses performed, including subgroup analyses and adjusted analyses, distinguishing pre-specified from exploratory	9-10
Harms	19	All important harms or unintended effects in each group (for specific guidance see CONSORT for harms)	11
Discussion			
Limitations	20	Trial limitations, addressing sources of potential bias, imprecision, and, if relevant, multiplicity of analyses	11
Generalisability	21	Generalisability (external validity, applicability) of the trial findings	10-11
Interpretation	22	Interpretation consistent with results, balancing benefits and harms, and considering other relevant evidence	10-11
Other information			
Registration	23	Registration number and name of trial registry	12
Protocol	24	Where the full trial protocol can be accessed, if available	No publication
Funding	25	Sources of funding and other support (such as supply of drugs), role of funders	12

*We strongly recommend reading this statement in conjunction with the CONSORT 2010 Explanation

and Elaboration for important clarifications on all the items. If relevant, we also recommend reading CONSORT extensions for cluster randomised trials, non-inferiority and equivalence trials, non-pharmacological treatments, herbal interventions, and pragmatic trials. Additional extensions are forthcoming: for those and for up to date references relevant to this checklist, see www.consort-statement.org.

Supplement Figure S1 Consort 2010 flow diagram

CONSORT

TRANSPARENT REPORTING of TRIALS

CONSORT 2010 Flow Diagram

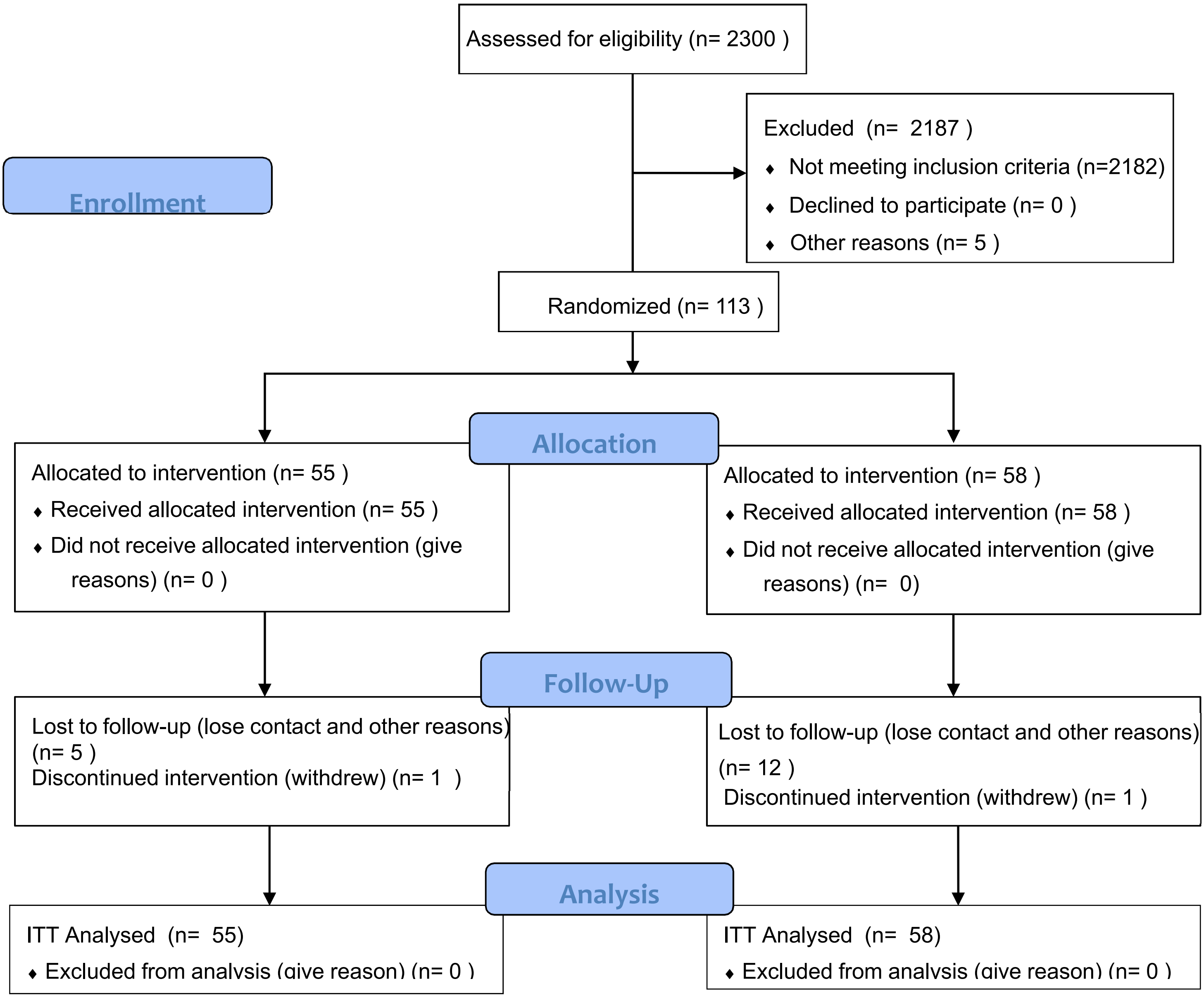

Supplement Figure S1 Consort 2010 flow diagram

Supplement Figure S2 Lipids between the intervention and control groups

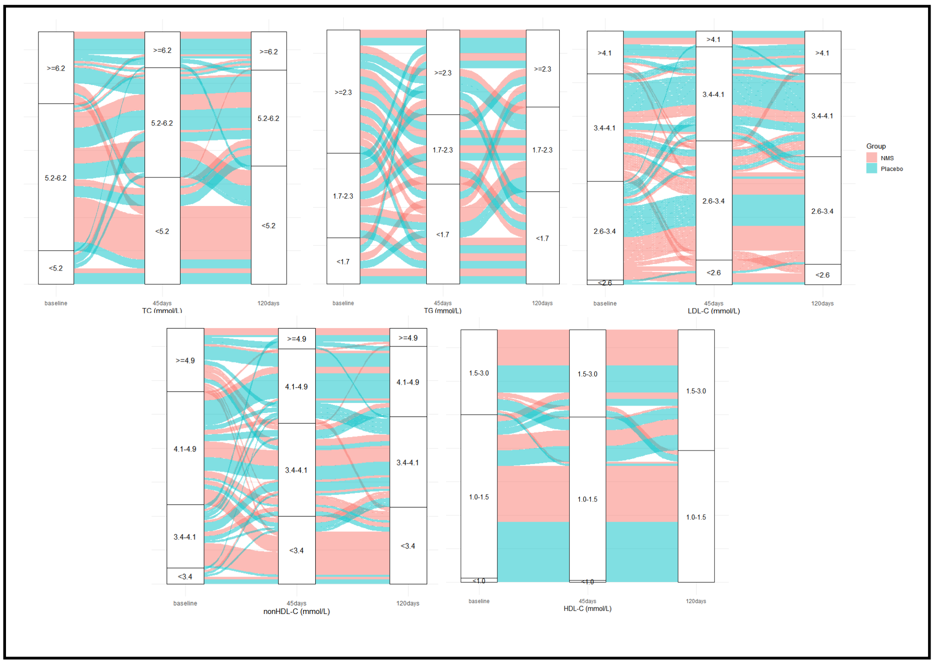

Supplement Figure S2 Lipids between the intervention and control groups

In the per-protocol population analysis, indicators remained consistent with previous conclusions (Supplement Table S3).

Supplement Table S3 Primary and second outcomes in the per-protocol population

End outcomes	NMS (*n*=48)	Placebo (*n*=44)	NMS vs. placebo	
	mean（95%CI)		mean difference (95%CI)	*P* value
Primary outcomes				
TC				
Baseline, mmol/L	5.98 (5.75 to 6.21)	5.92 (5.63 to 6.20)	0.07 (0.13 to 0.01)	0.642
45-days, mmol/L	4.96 (4.77 to 5.14) ***	5.44 (5.25 to 5.64) ***	-0.49 (-0.48 to -0.50)	<0.001
Absolute change from baseline to 45-days, mmol/L	-1.03 (-1.21 to -0.84)	-0.47 (-0.70 to -0.24)	-0.56 (-0.51 to -0.60)	<0.001
Percentage change from baseline to 45-days, %	-16.72 (-19.44 to -14.01)	-6.63 (-10.46 to -2.80)	-10.09 (-8.98 to -11.20)	<0.001
120-days, mmol/L	5.01 (4.78 to 5.24) ***	5.45 (5.26 to 5.64) ***	-0.44 (-0.49 to -0.40)	0.001
Absolute change from baseline to 120-days, mmol/L	-0.97 (-1.15 to -0.80)	-0.47 (-0.70 to -0.23)	-0.51 (-0.45 to -0.57)	0.001
Percentage change from baseline to 120-days, %	-16.11 (-18.81 to -13.41)	-6.56 (-10.10 to -3.03)	-9.54 (-8.71 to -10.38)	<0.001
TG				
Baseline, mmol/L	2.69 (2.36 to 3.03)	2.75 (2.42 to 3.08)	-0.06 (-0.06 to -0.05)	0.555
45-days, mmol/L	1.97 (1.65 to 2.30) ***	2.12 (1.90 to 2.34) ***	-0.15 (-0.26 to -0.05)	0.070
Absolute change from baseline to 45-days, mmol/L	-0.72 (-0.97 to -0.47)	-0.63 (-0.90 to -0.36)	-0.09 (-0.07 to -0.12)	0.529
Percentage change from baseline to 45-days, %	-25.06 (-32.39 to -17.73)	-17.28 (-26.21 to -8.36)	-7.78 (-6.19 to -9.38)	0.141
120-days, mmol/L	1.93 (1.70 to 2.17) ***	2.19 (1.96 to 2.42) ***	-0.26 (-0.26 to -0.25)	0.077

End outcomes	NMS (*n*=48)	Placebo (*n*=44)	NMS vs. placebo	
	mean（95%CI)		mean difference (95%CI)	*P* value
Primary outcomes				
Absolute change from baseline to 120-days, mmol/L	-0.76 (-1.00 to -0.52)	-0.56 (-0.79 to -0.33)	-0.20 (-0.21 to -0.19)	0.215
Percentage change from baseline to 120-days, %	-24.07 (-31.63 to -16.52)	-14.67 (-24.07 to -5.26)	-9.41 (-7.56 to -11.25)	0.098
LDL-C				
Baseline, mmol/L	3.66 (3.47 to 3.85)	3.60 (3.43 to 3.77)	0.06 (0.04 to 0.07)	0.746
45-days, mmol/L	3.04 (2.88 to 3.20) ***	3.52 (3.39 to 3.64)	-0.48 (-0.51 to -0.44)	<0.001
Absolute change from baseline to 45-days, mmol/L	-0.62 (-0.75 to -0.48)	-0.08 (-0.22 to 0.05)	-0.53 (-0.54 to -0.53)	<0.001
Percentage change from baseline to 45-days, %	-16.18 (-19.66 to -12.70)	-1.02 (-4.93 to 2.89)	-15.16 (-14.73 to -15.59)	<0.001
120-days, mmol/L	3.20 (2.99 to 3.42) ***	3.63 (3.47 to 3.78)	-0.42 (-0.48 to -0.36)	0.002
Absolute change from baseline to 120-days, mmol/L	-0.45 (-0.60 to -0.30)	0.03 (-0.12 to 0.17)	-0.48 (-0.48 to -0.47)	<0.001
Percentage change from baseline to 120-days, %	-12.20 (-16.31 to -8.08)	1.88 (-2.29 to 6.05)	-14.08 (-14.02 to -14.13)	<0.001
non-HDL-C				
Baseline, mmol/L	4.54 (4.31 to 4.78)	4.52 (4.26 to 4.77)	0.03 (0.05 to 0.01)	0.994
45-days, mmol/L	3.47 (3.28 to 3.66) ***	4.03 (3.87 to 4.19) ***	-0.56 (-0.59 to -0.53)	<0.001
Absolute change from baseline to 45-days, mmol/L	-1.08 (-1.25 to -0.90)	-0.49 (-0.69 to -0.29)	-0.59 (-0.56 to -0.61)	<0.001
Percentage change from baseline to 45-days, %	-23.23 (-26.47 to -19.99)	-9.15 (-13.33 to -4.97)	-14.08 (-13.13 to -15.02)	<0.001
120-days, mmol/L	3.46 (3.24 to 3.69) ***	3.93 (3.79 to 4.08) ***	-0.47 (-0.55 to -0.39)	0.001
Absolute change from baseline to 120-days, mmol/L	-1.08 (-1.25 to -0.91)	-0.58 (-0.80 to -0.36)	-0.50 (-0.45 to -0.55)	0.001
Percentage change from baseline to 120-days, %	-23.59 (-26.96 to -20.22)	-11.11 (-15.23 to -6.99)	-12.48 (-11.73 to -13.23)	<0.001
LDL-C to HDL-C ratio				
Baseline	2.62 (2.44 to 2.80)	2.63 (2.47 to 2.79)	-0.01 (-0.03 to 0.01)	0.849
45-days	2.11 (1.96 to 2.26) ***	2.55 (2.39 to 2.70) *	-0.44 (-0.44 to -0.44)	<0.001
Absolute change from baseline to 45-days	-0.51 (-0.60 to -0.42)	-0.08 (-0.15 to -0.01)	-0.43 (-0.45 to -0.41)	<0.001
Percentage change from baseline to 45-days, %	-19.18 (-22.28 to -16.07)	-2.73 (-5.50 to 0.05)	-16.45 (-16.78 to -16.12)	<0.001
120-days	2.13 (1.97 to 2.30) ***	2.46 (2.31 to 2.60) **	-0.33 (-0.34 to -0.31)	0.004
Absolute change from baseline to 120-days	-0.49 (-0.60 to -0.39)	-0.17 (-0.29 to -0.06)	-0.32 (-0.31 to -0.33)	<0.001
Percentage change from baseline to 120-days, %	-18.30 (-22.27 to -14.33)	-5.66 (-9.74 to -1.57)	-12.65 (-12.53 to -12.76)	<0.001
HDL-C				
Baseline, mmol/L	1.44 (1.36 to 1.52)	1.40 (1.32 to 1.48)	0.04 (0.04 to 0.04)	0.547
45-days, mmol/L	1.49 (1.41 to 1.57) **	1.42 (1.34 to 1.49)	0.07 (0.07 to 0.07)	0.254
Absolute change from baseline to 45-days, mmol/L	0.05 (0.01 to 0.09)	0.02 (-0.04 to 0.07)	0.03 (0.05 to 0.01)	0.173
Percentage change from baseline to 45-days, %	3.93 (1.24 to 6.63)	2.18 (-1.79 to 6.16)	1.75 (3.03 to 0.47)	0.143
120-days, mmol/L	1.54 (1.47 to 1.62) ***	1.52 (1.43 to 1.60) ***	0.03 (0.04 to 0.01)	0.514
Absolute change from baseline to 120-days, mmol/L	0.11 (0.06 to 0.15)	0.12 (0.06 to 0.17)	-0.01 (0.00 to -0.03)	0.729
Percentage change from baseline to 120-days, %	8.06 (5.04 to 11.09)	8.99 (5.00 to 12.99)	-0.93 (0.04 to -1.91)	0.708
Second outcomes				
Right CIMT				
Baseline, mm	0.82 (0.76 to 0.87)	0.81 (0.75 to 0.86)	0.01 (0.01 to 0.01)	0.815
120-days, mm	0.84 (0.79 to 0.88)	0.86 (0.81 to 0.92)	-0.03 (-0.02 to -0.03)	0.482
Absolute change from baseline to 120-days, mm	0.02 (-0.04 to 0.08)	0.06 (0.01 to 0.11)	-0.04 (-0.05 to -0.03)	0.216
Percentage change from baseline to 120-days, %	7.42 (-2.50 to 17.35)	9.99 (3.01 to 16.97)	-2.57 (-5.51 to 0.38)	0.227
Left CIMT				
Baseline, mm	0.86 (0.80 to 0.92)	0.90 (0.83 to 0.97)	-0.04 (-0.03 to -0.04)	0.574

End outcomes	NMS (*n*=48)	Placebo (*n*=44)	NMS vs. placebo	
	mean（95%CI)		mean difference (95%CI)	*P* value
Primary outcomes				
120-days, mm	0.88 (0.82 to 0.94)	0.90 (0.83 to 0.96)	-0.02 (-0.02 to -0.02)	0.677
Absolute change from baseline to 120-days, mm	0.01 (-0.02 to 0.05)	0.00 (-0.06 to 0.06)	0.02 (0.04 to 0.00)	0.783
Percentage change from baseline to 120-days, %	3.18 (-1.85 to 8.20)	2.21 (-4.31 to 8.73)	0.97 (2.46 to -0.53)	0.765
Average (Left and right) CIMT				
Baseline, mm	0.84 (0.79 to 0.89)	0.85 (0.80 to 0.91)	-0.01 (-0.01 to -0.01)	0.754
120-days, mm	0.85 (0.80 to 0.90)	0.88 (0.83 to 0.93)	-0.03 (-0.02 to -0.03)	0.502
Absolute change from baseline to 120-days, mm	0.01 (-0.03 to 0.05)	0.03 (-0.02 to 0.07)	-0.01 (-0.01 to -0.02)	0.490
Percentage change from baseline to 120-days, %	3.44 (-2.05 to 8.92)	4.88 (-0.57 to 10.32)	-1.44 (-1.48 to -1.40)	0.493

Mean difference refers to the disparity between two groups. The difference is considered statistically significant when P<0.05, denoted as *; when P<0.01, denoted as **; and when P<0.001, denoted as ***.

There were no significant differences between the two groups in terms of coagulation, liver function, renal function, blood glucose, blood routine, and physical examination (Supplement Table S4). At the 45-day intervention, there was a mean difference of 1.06 (1.05 to 1.07) in the absolute change of PT between the two groups.

Supplement Table S4 Laboratory values and physical examination of interest

Variables	NMS (*n*=55)	Placebo (*n*=58)	NMS vs. placebo	
	mean（95%CI)		mean difference (95%CI)	*P* value
Coagulation				
PT, S				
Baseline	10.35 (10.13 to 10.57)	11.00 (10.37 to 11.64)	-0.66 (-0.24 to -1.07)	0.454
45-days	11.63 (10.95 to 12.31)	11.23 (10.92 to 11.53)	0.41 (0.03 to 0.78)	0.247
Absolute change from baseline to 45-days	1.29 (0.63 to 1.94)	0.22 (-0.43 to 0.87)	1.06 (1.05 to 1.07)	0.019
120-days	12.28 (11.83 to 12.72)	11.98 (11.41 to 12.54)	0.30 (0.43 to 0.17)	0.484
Absolute change from baseline to 120-days	1.89 (1.42 to 2.36)	0.93 (0.07 to 1.80)	0.96 (1.36 to 0.56)	0.128
INR				
Baseline	0.88 (0.85 to 0.91)	0.88 (0.85 to 0.90)	0.00 (0.00 to 0.01)	0.879
45-days	0.88 (0.86 to 0.90)	0.89 (0.87 to 0.92)	-0.01 (-0.01 to -0.02)	0.268
Absolute change from baseline to 45-days	0.00 (-0.03 to 0.02)	0.01 (0.00 to 0.03)	-0.02 (-0.03 to -0.01)	0.381
120-days	0.89 (0.87 to 0.91)	0.89 (0.87 to 0.92)	0.00 (0.00 to -0.01)	0.730
Absolute change from baseline to 120-days	0.01 (-0.03 to 0.05)	0.02 (-0.01 to 0.04)	-0.01 (-0.02 to 0.00)	0.596
APTT, S				
Baseline	30.91 (29.50 to 32.31)	31.83 (30.15 to 33.51)	-0.93 (-0.66 to -1.20)	0.807
45-days	31.53 (30.56 to 32.51)	32.32 (30.64 to 34.01)	-0.79 (-0.08 to -1.50)	0.956
Absolute change from baseline to 45-days	0.63 (-0.84 to 2.09)	0.49 (-1.36 to 2.34)	0.14 (0.52 to -0.25)	0.863
120-days	32.29 (31.39 to 33.19)	32.69 (31.49 to 33.90)	-0.40 (-0.09 to -0.72)	0.707
Absolute change from baseline to 120-days	1.39 (0.11 to 2.67)	0.87 (-0.60 to 2.33)	0.52 (0.71 to 0.34)	0.910

Variables	NMS (*n*=55)	Placebo (*n*=58)	NMS vs. placebo	
	mean（95%CI)		mean difference (95%CI)	*P* value
Prothrombin time, S				
Baseline	10.85 (10.49 to 11.21)	10.79 (10.50 to 11.08)	0.06 (-0.02 to 0.13)	0.879
45-days	10.87 (10.66 to 11.09)	10.89 (10.43 to 11.36)	-0.02 (0.23 to -0.27)	0.257
Absolute change from baseline to 45-days	0.02 (-0.31 to 0.36)	0.10 (-0.31 to 0.51)	-0.08 (-0.01 to -0.15)	0.408
120-days	10.97 (10.72 to 11.22)	10.98 (10.68 to 11.29)	-0.02 (0.04 to -0.07)	0.726
Absolute change from baseline to 120-days	0.13 (-0.31 to 0.57)	0.23 (-0.06 to 0.53)	-0.10 (-0.25 to 0.04)	0.609
FIB, g/L				
Baseline	2.99 (2.83 to 3.15)	3.00 (2.79 to 3.20)	-0.01 (0.03 to -0.04)	0.511
45-days	2.97 (2.47 to 3.46)	2.87 (2.65 to 3.08)	0.10 (-0.18 to 0.38)	0.529
Absolute change from baseline to 45-days	-0.02 (-0.52 to 0.48)	-0.13 (-0.35 to 0.09)	0.11 (-0.18 to 0.39)	0.754
120-days	2.79 (2.65 to 2.92)	2.93 (2.58 to 3.27)	-0.14 (0.07 to -0.35)	0.630
Absolute change from baseline to 120-days	-0.18 (-0.34 to -0.02)	-0.05 (-0.39 to 0.29)	-0.13 (0.05 to -0.32)	0.682
Liver function				
Total bilirubin, μmol/L				
Baseline	17.79 (15.39 to 20.19)	18.87 (16.43 to 21.30)	-1.08 (-1.05 to -1.11)	0.453
45-days	18.16 (16.23 to 20.08)	19.04 (16.85 to 21.22)	-0.88 (-0.63 to -1.14)	0.796
120-days	16.18 (14.31 to 18.05)	17.04 (14.86 to 19.22)	-0.86 (-0.55 to -1.17)	0.886
Direct bilirubin, μmol/L				
Baseline	3.53 (3.12 to 3.94)	4.05 (3.59 to 4.52)	-0.53 (-0.47 to -0.59)	0.114
45-days	4.42 (4.04 to 4.80)	4.34 (3.90 to 4.77)	0.08 (0.13 to 0.03)	0.453
120-days	4.14 (3.77 to 4.51)	3.97 (3.60 to 4.34)	0.17 (0.16 to 0.17)	0.478
Indirect bilirubin, μmol/L				
Baseline	14.26 (12.07 to 16.45)	14.81 (12.62 to 17.01)	-0.55 (-0.55 to -0.55)	0.636
45-days	13.74 (12.12 to 15.35)	14.70 (12.89 to 16.52)	-0.97 (-0.76 to -1.17)	0.591
120-days	12.04 (10.46 to 13.62)	13.07 (11.19 to 14.94)	-1.03 (-0.73 to -1.32)	0.669
ALT, U/L				
Baseline	24.88 (20.75 to 29.01)	28.05 (23.23 to 32.87)	-3.17 (-2.48 to -3.86)	0.351
45-days	26.71 (23.25 to 30.17)	29.78 (24.01 to 35.54)	-3.07 (-0.76 to -5.37)	0.646
120-days	24.81 (21.29 to 28.33)	25.37 (20.54 to 30.19)	-0.56 (0.75 to -1.86)	0.662
AST, U/L				
Baseline	26.35 (22.11 to 30.58)	26.01 (23.62 to 28.39)	0.34 (-1.51 to 2.19)	0.759
45-days	26.04 (23.93 to 28.14)	26.99 (24.47 to 29.51)	-0.95 (-0.54 to -1.36)	0.961
120-days	25.74 (23.48 to 28.01)	24.69 (22.75 to 26.64)	1.05 (0.73 to 1.37)	0.571
AST/ALT				
Baseline	1.21 (1.09 to 1.34)	1.09 (0.99 to 1.18)	0.12 (0.09 to 0.16)	0.169
45-days	1.08 (0.99 to 1.17)	1.12 (1.02 to 1.23)	-0.04 (-0.03 to -0.05)	0.513
120-days	1.18 (1.08 to 1.28)	1.16 (1.06 to 1.27)	0.02 (0.02 to 0.01)	0.742
γ-GT, U/L				
Baseline	36.01 (28.30 to 43.73)	33.11 (26.87 to 39.35)	2.90 (1.43 to 4.38)	0.803
45-days	34.86 (28.05 to 41.67)	36.48 (27.13 to 45.83)	-1.62 (0.92 to -4.16)	0.868
120-days	33.45 (26.52 to 40.37)	32.36 (26.25 to 38.47)	1.09 (0.27 to 1.90)	0.836
ALP, U/L				
Baseline	77.87 (70.02 to 85.73)	80.25 (72.87 to 87.63)	-2.38 (-2.86 to -1.90)	0.694
45-days	72.84 (66.22 to 79.46)	80.53 (73.75 to 87.31)	-7.69 (-7.52 to -7.85)	0.085
120-days	91.71 (84.76 to 98.65)	96.57 (89.50 to 103.65)	-4.87 (-4.74 to -5.00)	0.327

Variables	NMS (*n*=55)	Placebo (*n*=58)	NMS vs. placebo	
	mean（95%CI)		mean difference (95%CI)	*P* value
TP, g/L				
Baseline	81.13 (80.26 to 82.00)	81.08 (80.03 to 82.13)	0.05 (0.24 to -0.13)	0.938
45-days	75.28 (72.66 to 77.89)	77.78 (76.83 to 78.73)	-2.50 (-4.17 to -0.84)	0.076
120-days	78.58 (77.71 to 79.45)	79.67 (78.66 to 80.68)	-1.09 (-0.96 to -1.23)	0.103
ALB, g/L				
Baseline	51.75 (51.21 to 52.28)	51.62 (51.05 to 52.19)	0.13 (0.16 to 0.09)	0.746
45-days	47.21 (46.57 to 47.86)	47.73 (46.96 to 48.49)	-0.51 (-0.39 to -0.63)	0.459
120-days	48.73 (48.15 to 49.32)	49.01 (48.31 to 49.71)	-0.28 (-0.17 to -0.40)	0.536
GLB, g/L				
Baseline	29.38 (28.56 to 30.20)	29.46 (28.55 to 30.36)	-0.07 (0.01 to -0.16)	0.905
45-days	29.37 (28.57 to 30.17)	30.05 (29.18 to 30.92)	-0.68 (-0.61 to -0.75)	0.252
120-days	29.84 (29.02 to 30.65)	30.66 (29.69 to 31.63)	-0.82 (-0.67 to -0.97)	0.379
ALB/GLB				
Baseline	1.78 (1.73 to 1.84)	1.78 (1.72 to 1.84)	0.00 (0.01 to 0.00)	0.935
45-days	1.63 (1.57 to 1.68)	1.61 (1.55 to 1.67)	0.02 (0.02 to 0.02)	0.714
120-days	1.65 (1.59 to 1.70)	1.62 (1.56 to 1.68)	0.03 (0.03 to 0.02)	0.725
Renal function				
Urea, mmol/L				
Baseline	6.28 (5.72 to 6.85)	6.39 (5.98 to 6.81)	-0.11 (-0.26 to 0.04)	0.760
45-days	5.17 (4.73 to 5.62)	5.15 (4.76 to 5.54)	0.02 (-0.04 to 0.08)	0.890
120-days	5.05 (4.67 to 5.43)	4.97 (4.56 to 5.37)	0.08 (0.11 to 0.06)	0.627
Uric acid, μmol/L				
Baseline	293.48 (266.70 to 320.27)	287.11 (262.23 to 311.99)	6.38 (4.47 to 8.28)	0.581
45-days	274.17 (252.22 to 296.11)	284.75 (261.25 to 308.25)	-10.58 (-9.03 to -12.14)	0.827
120-days	267.74 (244.66 to 290.82)	269.59 (245.22 to 293.97)	-1.85 (-0.56 to -3.14)	0.783
Blood glucose				
Fasting glucose, mmol/L				
Baseline	5.58 (5.14 to 6.02)	5.72 (5.29 to 6.15)	-0.14 (-0.15 to -0.13)	0.498
45-days	5.28 (4.86 to 5.70)	5.46 (5.02 to 5.91)	-0.19 (-0.16 to -0.21)	0.352
120-days	5.47 (5.00 to 5.94)	5.75 (5.26 to 6.24)	-0.28 (-0.26 to -0.31)	0.153
Blood routine				
White blood cell,10^9/L				
Baseline	6.18 (5.77 to 6.58)	6.11 (5.79 to 6.44)	0.06 (-0.02 to 0.14)	1.000
45-days	6.30 (5.86 to 6.73)	6.24 (5.77 to 6.70)	0.06 (0.09 to 0.03)	0.650
120-days	6.07 (5.64 to 6.51)	6.08 (5.71 to 6.45)	-0.01 (-0.08 to 0.06)	0.798
ANC,10^9/L				
Baseline	3.54 (3.20 to 3.87)	3.56 (3.33 to 3.79)	-0.03 (-0.12 to 0.07)	0.420
45-days	3.68 (3.32 to 4.03)	3.61 (3.24 to 3.99)	0.07 (0.08 to 0.05)	0.781
120-days	3.44 (3.10 to 3.77)	3.48 (3.20 to 3.76)	-0.05 (-0.10 to 0.01)	0.473
Neutrophil percentage, %				
Baseline	56.40 (54.26 to 58.53)	57.92 (56.15 to 59.68)	-1.52 (-1.89 to -1.15)	0.275
45-days	57.39 (55.26 to 59.52)	56.94 (54.97 to 58.91)	0.45 (0.29 to 0.61)	0.756
120-days	55.68 (53.70 to 57.65)	56.66 (54.82 to 58.49)	-0.98 (-1.12 to -0.84)	0.468
Absolute lymphocyte,10^9/L				
Baseline	2.17 (2.01 to 2.32)	2.06 (1.92 to 2.19)	0.11 (0.09 to 0.13)	0.255
45-days	2.15 (2.02 to 2.29)	2.13 (1.98 to 2.27)	0.03 (0.04 to 0.01)	0.411
120-days	2.18 (2.04 to 2.33)	2.11 (1.99 to 2.24)	0.07 (0.05 to 0.09)	0.483

Variables	NMS (*n*=55)	Placebo (*n*=58)	NMS vs. placebo	
	mean（95%CI)		mean difference (95%CI)	*P* value
Lymphocyte percentage, %				
Baseline	35.93 (33.80 to 38.06)	33.91 (32.29 to 35.53)	2.02 (1.50 to 2.53)	0.134
45-days	35.27 (33.15 to 37.40)	34.99 (33.18 to 36.80)	0.28 (-0.03 to 0.60)	0.838
120-days	36.83 (34.82 to 38.83)	35.39 (33.65 to 37.12)	1.44 (1.16 to 1.72)	0.280
AMC,10^9/L				
Baseline	0.34 (0.30 to 0.37)	0.35 (0.32 to 0.37)	-0.01 (-0.02 to 0.00)	0.208
45-days	0.34 (0.31 to 0.37)	0.34 (0.31 to 0.36)	0.00 (-0.01 to 0.01)	0.698
120-days	0.32 (0.29 to 0.35)	0.33 (0.31 to 0.36)	-0.01 (-0.02 to 0.00)	0.488
Percentage sofmonocytes, %				
Baseline	5.46 (5.07 to 5.86)	5.76 (5.42 to 6.11)	-0.30 (-0.35 to -0.25)	0.147
45-days	5.38 (5.04 to 5.71)	5.53 (5.22 to 5.84)	-0.15 (-0.17 to -0.13)	0.436
120-days	5.36 (4.98 to 5.73)	5.51 (5.23 to 5.79)	-0.15 (-0.25 to -0.06)	0.248
Eosinophil count,10^9/L				
Baseline	0.11 (0.08 to 0.14)	0.12 (0.10 to 0.14)	-0.01 (-0.01 to 0.00)	0.270
45-days	0.10 (0.08 to 0.12)	0.13 (0.10 to 0.16)	-0.03 (-0.02 to -0.04)	0.186
120-days	0.11 (0.08 to 0.13)	0.12 (0.10 to 0.14)	-0.01 (-0.02 to -0.01)	0.315
Immature granulocyte, n				
Baseline	0.01 (0.01 to 0.01)	0.01 (0.01 to 0.01)	0.00 (0.00 to 0.00)	0.193
45-days	0.01 (0.01 to 0.01)	0.01 (0.01 to 0.02)	0.00 (0.00 to -0.01)	0.797
120-days	0.01 (0.00 to 0.01)	0.01 (0.01 to 0.01)	0.00 (0.00 to -0.01)	0.305
Immature granulocyte percentage, %				
Baseline	0.15 (0.11 to 0.19)	0.16 (0.12 to 0.20)	-0.01 (-0.01 to -0.01)	0.809
45-days	0.18 (0.08 to 0.29)	0.15 (0.11 to 0.19)	0.03 (-0.03 to 0.10)	0.968
120-days	0.12 (0.10 to 0.14)	0.14 (0.10 to 0.19)	-0.02 (0.00 to -0.05)	0.616
Hemoglobin, g/L				
Baseline	144.85 (140.80 to 148.91)	148.34 (144.62 to 152.07)	-3.49 (-3.82 to -3.16)	0.206
45-days	145.51 (141.79 to 149.23)	146.10 (140.70 to 151.51)	-0.59 (1.09 to -2.28)	0.421
120-days	149.40 (145.80 to 153.00)	150.40 (146.32 to 154.47)	-1.00 (-0.52 to -1.47)	0.714
Mean hemoglobin, pg				
Baseline	31.13 (30.62 to 31.64)	31.17 (30.78 to 31.55)	-0.04 (-0.16 to 0.09)	0.975
45-days	31.08 (30.63 to 31.53)	31.05 (30.61 to 31.49)	0.03 (0.02 to 0.05)	0.954
120-days	31.19 (30.76 to 31.63)	31.16 (30.69 to 31.63)	0.03 (0.06 to 0.00)	0.972
Mean hemoglobin concentration, g/l				
Baseline	331.85 (329.95 to 333.76)	332.57 (331.10 to 334.04)	-0.71 (-1.14 to -0.28)	0.553
45-days	331.42 (330.03 to 332.81)	332.40 (330.81 to 333.98)	-0.98 (-0.78 to -1.17)	0.067
120-days	332.44 (330.92 to 333.96)	333.19 (331.44 to 334.94)	-0.75 (-0.52 to -0.99)	0.299
Red blood cell count,10^12/L				
Baseline	4.66 (4.54 to 4.78)	4.76 (4.65 to 4.88)	-0.11 (-0.11 to -0.10)	0.211
45-days	4.69 (4.58 to 4.80)	4.77 (4.65 to 4.89)	-0.08 (-0.07 to -0.08)	0.339
120-days	4.79 (4.68 to 4.90)	4.83 (4.71 to 4.96)	-0.04 (-0.03 to -0.06)	0.620
Hematocrit, %				
Baseline	43.63 (42.48 to 44.79)	44.58 (43.50 to 45.67)	-0.95 (-1.02 to -0.88)	0.230
45-days	43.87 (42.81 to 44.94)	44.47 (43.35 to 45.59)	-0.60 (-0.54 to -0.65)	0.441
120-days	44.90 (43.87 to 45.92)	45.13 (43.96 to 46.30)	-0.23 (-0.09 to -0.38)	0.766
MCV, fL				
Baseline	93.79 (92.45 to 95.13)	93.71 (92.63 to 94.79)	0.08 (-0.18 to 0.33)	0.623
45-days	93.77 (92.54 to 94.99)	91.85 (88.48 to 95.21)	1.92 (4.06 to -0.22)	0.509

Variables	NMS (*n*=55)	Placebo (*n*=58)	NMS vs. placebo	
	mean（95%CI)		mean difference (95%CI)	*P* value
120-days	93.82 (92.65 to 94.99)	93.50 (92.32 to 94.67)	0.32 (0.33 to 0.32)	0.629
RDW-CV, %				
Baseline	13.41 (13.08 to 13.73)	13.31 (13.13 to 13.50)	0.09 (-0.05 to 0.23)	0.388
45-days	13.28 (12.94 to 13.62)	13.27 (13.08 to 13.46)	0.01 (-0.15 to 0.16)	0.080
120-days	12.99 (12.73 to 13.24)	13.19 (12.92 to 13.46)	-0.20 (-0.18 to -0.22)	0.285
PLT,10^9/L				
Baseline	226.40 (209.80 to 243.00)	220.43 (204.98 to 235.88)	5.97 (4.82 to 7.12)	0.482
45-days	234.31 (216.46 to 252.16)	222.88 (207.12 to 238.63)	11.43 (9.33 to 13.53)	0.273
120-days	227.24 (210.03 to 244.44)	227.53 (212.01 to 243.06)	-0.30 (-1.98 to 1.38)	0.920
PCT, %				
Baseline	0.23 (0.21 to 0.25)	0.22 (0.21 to 0.24)	0.01 (0.00 to 0.01)	0.585
45-days	0.24 (0.22 to 0.25)	0.23 (0.22 to 0.24)	0.01 (0.00 to 0.01)	0.425
120-days	0.23 (0.22 to 0.25)	0.23 (0.22 to 0.24)	0.00 (0.00 to 0.01)	0.872
MPV, fL				
Baseline	10.25 (9.98 to 10.51)	10.39 (10.04 to 10.74)	-0.15 (-0.06 to -0.23)	0.908
45-days	10.15 (9.90 to 10.40)	10.27 (9.77 to 10.77)	-0.12 (0.13 to -0.37)	0.650
120-days	10.36 (10.08 to 10.64)	10.33 (9.98 to 10.69)	0.03 (0.11 to -0.04)	0.885
PDW, fL				
Baseline	16.25 (16.17 to 16.33)	16.28 (16.20 to 16.36)	-0.03 (-0.03 to -0.03)	0.550
45-days	16.22 (16.13 to 16.30)	16.29 (16.20 to 16.38)	-0.07 (-0.06 to -0.08)	0.244
120-days	16.30 (16.22 to 16.38)	16.27 (16.18 to 16.36)	0.03 (0.04 to 0.01)	0.645
P-LCR,10^9/L				
Baseline	62.29 (57.40 to 67.18)	60.50 (56.64 to 64.36)	1.79 (0.76 to 2.82)	0.566
45-days	62.29 (57.71 to 66.87)	61.69 (57.28 to 66.10)	0.60 (0.43 to 0.77)	0.711
120-days	64.22 (59.03 to 69.40)	62.34 (58.08 to 66.61)	1.87 (0.95 to 2.80)	0.577
Physical Examination of Interest				
BMI, kg/m²				
Baseline	26.17 (25.43 to 26.91)	26.61 (25.84 to 27.38)	-0.44 (-0.41 to -0.47)	0.412
45-days	26.27 (25.53 to 27.02)	26.65 (25.88 to 27.43)	-0.38 (-0.35 to -0.41)	0.483
120-days	26.38 (25.65 to 27.12)	26.66 (25.91 to 27.41)	-0.28 (-0.26 to -0.29)	0.596
SBP, mmHg				
Baseline	132.09 (127.47 to 136.71)	132.71 (127.98 to 137.43)	-0.62 (-0.51 to -0.72)	0.852
45-days	130.16 (125.95 to 134.38)	130.66 (125.92 to 135.39)	-0.49 (0.02 to -1.01)	0.877
120-days	133.47 (129.23 to 137.71)	133.90 (129.87 to 137.92)	-0.42 (-0.64 to -0.21)	0.885
DBP, mmHg				
Baseline	85.07 (82.15 to 87.99)	83.47 (80.71 to 86.22)	1.61 (1.44 to 1.77)	0.424
45-days	84.56 (82.08 to 87.05)	84.14 (81.15 to 87.12)	0.43 (0.93 to -0.08)	0.861
120-days	84.89 (82.44 to 87.34)	83.66 (81.19 to 86.12)	1.24 (1.25 to 1.22)	0.477
Waist-hip ratio				
Baseline	93.54 (92.09 to 94.99)	94.18 (92.53 to 95.83)	-0.64 (-0.44 to -0.84)	0.561
45-days	91.49 (90.05 to 92.93)	91.45 (90.11 to 92.78)	0.05 (-0.06 to 0.15)	0.982
120-days	90.77 (89.06 to 92.48)	90.56 (88.89 to 92.23)	0.21 (0.17 to 0.26)	0.859

P value means the comparison of the two groups in the same period; PT: prothrombin time, INR: international normalized ratio, APTT: activated partial prothrombin time, FIB: Fibrinogen, AST: aspartate aminotransferase, ALT: alanine aminotransferase, γ-GT: γ-glutamyl transpeptidase, ALP: alkaline phosphatase, TP: total protein, ALB: albumin, GLB: globulin, ANC: absolute neutrophil count, AMC:

absolute monocyte count, MCV: mean corpuscular volume, RDW-CV: red blood cell volume distribution width coefficient of variation, PLT: platelet, PDW: Platelet distribution width, MPV: mean platelet volume, PCT: MPV*PDW, P-LCR: platelet-large cell ratio; BMI: body mass index, SBP: systolic blood pressure, DBP: diastolic blood pressure.

At baseline, there were no significant differences in the consumption of milk, dairy and soy nuts products, plant foods, cereals and potatoes, vegetables, fruit, and metabolic levels between the two groups. These similarities persisted at the 45-day and 120-day marks (Supplement Table S5).

Supplement Table S5 Dietary intakes and metabolic levels at baseline, 45-days, and 120-days

Variables	NMS (*n*=56)	Placebo (*n*=58)	NMS vs. placebo	
	mean（95%CI)		mean difference (95%CI)	*P* value
Milk, dairy and soy nuts products, times/day				
Baseline	0.80 (0.58 to 1.03)	0.76 (0.58 to 0.94)	0.04 (-0.01 to 0.09)	0.724
45-days	1.00 (0.73 to 1.27)	0.81 (0.63 to 0.99)	0.19 (0.10 to 0.27)	0.633
120-days	1.09 (0.83 to 1.35)	0.87 (0.69 to 1.05)	0.22 (0.14 to 0.30)	0.448
Animal foods, times/day				
Baseline	1.22 (0.95 to 1.48)	1.34 (1.06 to 1.63)	-0.13 (-0.11 to -0.15)	0.609
45-days	1.18 (0.94 to 1.41)	1.29 (1.01 to 1.57)	-0.12 (-0.07 to -0.16)	0.841
120-days	1.37 (1.08 to 1.65)	1.30 (1.05 to 1.56)	0.06 (0.03 to 0.09)	0.743
Plant foods, times/day				
Baseline	7.37 (6.58 to 8.16)	7.38 (6.73 to 8.03)	-0.01 (-0.15 to 0.12)	0.886
45-days	6.91 (6.15 to 7.66)	6.77 (6.07 to 7.47)	0.14 (0.09 to 0.19)	0.788
120-days	6.56 (5.92 to 7.20)	6.73 (6.17 to 7.28)	-0.16 (-0.24 to -0.08)	0.483
Cereals and potatoes, times/day				
Baseline	3.70 (3.35 to 4.05)	3.49 (3.22 to 3.76)	0.21 (0.13 to 0.29)	0.499
45-days	3.50 (3.16 to 3.83)	3.21 (2.93 to 3.49)	0.29 (0.23 to 0.35)	0.411
120-days	3.29 (3.00 to 3.57)	3.14 (2.89 to 3.40)	0.14 (0.11 to 0.17)	0.639
Vegetables and fruit, times/day				
Baseline	3.67 (3.07 to 4.26)	3.89 (3.38 to 4.40)	-0.22 (-0.31 to -0.14)	0.629
45-days	3.41 (2.79 to 4.03)	3.56 (3.03 to 4.10)	-0.15 (-0.23 to -0.07)	0.514
120-days	3.28 (2.80 to 3.76)	3.58 (3.11 to 4.05)	-0.30 (-0.31 to -0.29)	0.274
Metabolic level, kcal				
Baseline	2132.78 (1880.76 to 2384.79)	1954.52 (1785.49 to 2123.56)	178.25 (95.27 to 261.23)	0.526
45-days	2142.08 (1886.74 to 2397.41)	1959.50 (1788.66 to 2130.34)	182.57 (98.08 to 267.07)	0.579
120-days	2170.68 (1921.59 to 2419.76)	1959.42 (1789.32 to 2129.53)	211.26 (132.28 to 290.23)	0.369

P value means the comparison of the two groups in the same period; Metabolic level: weight * MET*24h, Calculated as physical activity level based on MET (exercise equivalents), referring to the 2011 Japanese exercise standard: sleeping for 1 MET; eating for 1.5 METS; computer for 1.5 METS; TV for 1.0 METS; resting for 1.3 METS; housework for 2.0 METS; light standing work for 2.3 METS; medium-heavy standing

work for 4.5 METS; light walking work for 3.5 METS; moderate-heavy walking for 4.5 METS; cycling for 3.5 METS; driving for 1.5 METS; ; dancing for 7 METSs; other moderate physical activity for 5 METS.

In patients with borderline mixed hyperlipidemia, for TC values ranging from 5.2 to 6.2 and TG values from 1.7 to 2.3, the findings were in line with prior analyses. However, there were exceptions: TC initial values varied at the first follow-up, and while TG raw values, the differences and percentages showed variation (Supplement Table S6).

Supplement Table S6 Primary and second outcomes in the borderline hyperlipidaemia.

End outcomes	NMS (*n*=26)	Placebo (*n*=21)	NMS vs. placebo	
	mean（95%CI)		mean difference (95%CI)	*P* value
Primary outcomes				
TC				
Baseline, mmol/L	5.86 (5.64 to 6.08)	5.60 (5.21 to 6.00)	0.25 (0.44 to 0.07)	0.700
45-days, mmol/L	4.90 (4.65 to 5.15) ***	5.20 (4.87 to 5.52) **	-0.30 (-0.22 to -0.37)	0.141
Absolute change from baseline to 45-days, mmol/L	-0.96 (-1.20 to -0.72)	-0.41 (-0.70 to -0.11)	-0.55 (-0.50 to -0.60)	0.004
Percentage change from baseline to 45-days, %	-16.11 (-19.92 to -12.31)	-6.24 (-11.26 to -1.22)	-9.87 (-8.66 to -11.08)	0.002
120-days, mmol/L	4.94 (4.66 to 5.23) ***	5.41 (5.08 to 5.73)	-0.46 (-0.42 to -0.51)	0.032
Absolute change from baseline to 120-days, mmol/L	-0.91 (-1.16 to -0.67)	-0.20 (-0.44 to 0.05)	-0.72 (-0.72 to -0.71)	<0.001
Percentage change from baseline to 120-days, %	-15.50 (-19.36 to -11.64)	-2.66 (-7.08 to 1.77)	-12.84 (-12.28 to -13.40)	<0.001
TG				
Baseline, mmol/L	2.21 (1.91 to 2.51)	2.01 (1.77 to 2.26)	0.20 (0.14 to 0.25)	0.608
45-days, mmol/L	1.61 (1.33 to 1.89) ***	1.83 (1.52 to 2.13)	-0.22 (-0.19 to -0.24)	0.227
Absolute change from baseline to 45-days, mmol/L	-0.60 (-0.89 to -0.30)	-0.19 (-0.45 to 0.08)	-0.41 (-0.44 to -0.38)	0.036
Percentage change from baseline to 45-days, %	-24.67 (-35.33 to -14.00)	-6.91 (-20.42 to 6.61)	-17.76 (-14.91 to -20.61)	0.025
120-days, mmol/L	1.63 (1.41 to 1.85) ***	1.93 (1.71 to 2.14)	-0.29 (-0.30 to -0.29)	0.051
Absolute change from baseline to 120-days, mmol/L	-0.58 (-0.86 to -0.29)	-0.08 (-0.30 to 0.13)	-0.49 (-0.56 to -0.42)	0.010
Percentage change from baseline to 120-days, %	-22.35 (-32.69 to -12.00)	1.00 (-15.43 to 17.44)	-23.35 (-17.26 to -29.44)	0.010
LDL-C				
Baseline, mmol/L	3.64 (3.49 to 3.79)	3.49 (3.16 to 3.82)	0.15 (0.32 to -0.03)	0.764
45-days, mmol/L	3.01 (2.83 to 3.19) ***	3.40 (3.13 to 3.67)	-0.39 (-0.29 to -0.48)	0.018
Absolute change from baseline to 45-days, mmol/L	-0.63 (-0.79 to -0.47)	-0.09 (-0.26 to 0.08)	-0.53 (-0.53 to -0.54)	<0.001
Percentage change from baseline to 45-days, %	-17.08 (-21.23 to -12.94)	-1.14 (-6.44 to 4.16)	-15.95 (-14.79 to -17.10)	<0.001
120-days, mmol/L	3.14 (2.91 to 3.36) ***	3.59 (3.29 to 3.89)	-0.45 (-0.39 to -0.52)	0.016
Absolute change from baseline to 120-days, mmol/L	-0.50 (-0.71 to -0.29)	0.10 (-0.10 to 0.30)	-0.60 (-0.61 to -0.59)	<0.001
Percentage change from baseline to 120-days, %	-13.65 (-19.19 to -8.12)	4.43 (-1.54 to 10.40)	-18.08 (-17.64 to -18.52)	<0.001
non-HDL-C				
Baseline, mmol/L	4.38 (4.16 to 4.59)	4.21 (3.84 to 4.57)	0.17 (0.32 to 0.02)	0.417
45-days, mmol/L	3.38 (3.15 to 3.61) ***	3.82 (3.52 to 4.11) **	-0.44 (-0.38 to -0.50)	0.019

End outcomes	NMS (*n*=26)	Placebo (*n*=21)	NMS vs. placebo	
	mean（95%CI)		mean difference (95%CI)	*P* value
Absolute change from baseline to 45-days, mmol/L	-1.00 (-1.21 to -0.79)	-0.39 (-0.64 to -0.14)	-0.61 (-0.57 to -0.64)	<0.001
Percentage change from baseline to 45-days, %	-22.61 (-26.99 to -18.23)	-7.89 (-13.55 to -2.23)	-14.72 (-13.44 to -16.00)	<0.001
120-days, mmol/L	3.34 (3.08 to 3.61) ***	3.89 (3.60 to 4.19) *	-0.55 (-0.52 to -0.58)	0.007
Absolute change from baseline to 120-days, mmol/L	-1.03 (-1.27 to -0.79)	-0.32 (-0.56 to -0.07)	-0.72 (-0.71 to -0.72)	<0.001
Percentage change from baseline to 120-days, %	-23.47 (-28.33 to -18.61)	-6.10 (-11.74 to -0.45)	-17.37 (-16.59 to -18.16)	<0.001
LDL-C to HDL-C ratio				
Baseline	2.54 (2.32 to 2.76)	2.61 (2.23 to 2.99)	-0.07 (0.09 to -0.24)	0.874
45-days	2.04 (1.85 to 2.23) ***	2.57 (2.22 to 2.93)	-0.54 (-0.37 to -0.70)	0.009
Absolute change from baseline to 45-days	-0.50 (-0.60 to -0.41)	-0.04 (-0.15 to 0.08)	-0.46 (-0.45 to -0.48)	<0.001
Percentage change from baseline to 45-days, %	-19.70 (-23.11 to -16.28)	-0.11 (-4.97 to 4.75)	-19.59 (-18.14 to -21.04)	<0.001
120-days	2.02 (1.82 to 2.22) ***	2.47 (2.15 to 2.80)	-0.45 (-0.33 to -0.58)	0.011
Absolute change from baseline to 120-days	-0.52 (-0.65 to -0.39)	-0.14 (-0.33 to 0.05)	-0.38 (-0.32 to -0.44)	0.001
Percentage change from baseline to 120-days, %	-20.38 (-25.17 to -15.60)	-3.12 (-9.96 to 3.72)	-17.26 (-15.20 to -19.32)	<0.001
HDL-C				
Baseline, mmol/L	1.48 (1.37 to 1.60)	1.40 (1.26 to 1.53)	0.09 (0.11 to 0.07)	0.322
45-days, mmol/L	1.52 (1.41 to 1.64)	1.38 (1.24 to 1.52)	0.14 (0.17 to 0.12)	0.106
Absolute change from baseline to 45-days, mmol/L	0.04 (-0.01 to 0.10)	-0.02 (-0.10 to 0.07)	0.06 (0.09 to 0.03)	0.256
Percentage change from baseline to 45-days, %	3.38 (-0.36 to 7.12)	-0.41 (-5.93 to 5.11)	3.79 (5.57 to 2.01)	0.202
120-days, mmol/L	1.60 (1.49 to 1.71) ***	1.51 (1.37 to 1.66) **	0.09 (0.12 to 0.05)	0.336
Absolute change from baseline to 120-days, mmol/L	0.12 (0.08 to 0.16)	0.12 (0.03 to 0.20)	0.00 (0.04 to -0.04)	0.764
Percentage change from baseline to 120-days, %	8.58 (5.49 to 11.68)	9.17 (3.12 to 15.22)	-0.59 (2.36 to -3.54)	0.864
Second outcomes				
Right CIMT				
Baseline, mm	0.76 (0.69 to 0.83)	0.79 (0.70 to 0.89)	-0.03 (-0.01 to -0.06)	0.712
120-days, mm	0.81 (0.75 to 0.87)	0.77 (0.70 to 0.85)	0.04 (0.05 to 0.03)	0.351
Absolute change from baseline to 120-days, mm	0.05 (-0.02 to 0.12)	-0.02 (-0.10 to 0.06)	0.07 (0.08 to 0.07)	0.080
Percentage change from baseline to 120-days, %	12.06 (-3.42 to 27.54)	1.39 (-9.94 to 12.71)	10.68 (6.53 to 14.83)	0.078
Left CIMT				
Baseline, mm	0.87 (0.79 to 0.96)	0.88 (0.77 to 1.00)	-0.01 (0.02 to -0.04)	0.837
120-days, mm	0.89 (0.81 to 0.97)	0.86 (0.75 to 0.96)	0.03 (0.05 to 0.01)	0.627
Absolute change from baseline to 120-days, mm	0.02 (-0.03 to 0.06)	-0.02 (-0.07 to 0.02)	0.04 (0.04 to 0.04)	0.322
Percentage change from baseline to 120-days, %	2.77 (-2.93 to 8.48)	-1.49 (-6.57 to 3.60)	4.26 (3.64 to 4.88)	0.336
Average (Left and right) CIMT				
Baseline, mm	0.82 (0.75 to 0.88)	0.84 (0.74 to 0.93)	-0.02 (0.01 to -0.05)	0.863
120-days, mm	0.85 (0.78 to 0.91)	0.81 (0.73 to 0.90)	0.03 (0.05 to 0.02)	0.539
Absolute change from baseline to 120-days, mm	0.03 (-0.02 to 0.08)	-0.02 (-0.07 to 0.03)	0.05 (0.05 to 0.05)	0.154
Percentage change from baseline to 120-days, %	4.86 (-1.80 to 11.52)	-1.38 (-6.83 to 4.07)	6.24 (5.03 to 7.46)	0.161

Mean difference refers to the disparity between two groups. The difference is considered statistically significant when P<0.05, denoted as *; when P<0.01, denoted as **; and when P<0.001, denoted as ***.

Subgroup analyses by age for those aged 30-60 years, those aged 60 years and older were generally consistent with the previous results (Supplement Table S7).

Supplement Table S7 Primary and second outcomes for those aged 30-60 and over 60 years

End outcomes	NMS	Placebo	NMS vs. placebo	
	mean（95%CI)		mean difference (95%CI)	*P* value
30-60 years	NMS (*n*=26)	Placebo (*n*=26)		
Primary outcomes				
TC				
Baseline, mmol/L	6.00 (5.72 to 6.28)	5.93 (5.65 to 6.22)	0.07 (0.07 to 0.07)	0.729
45-days, mmol/L	4.88 (4.57 to 5.19)	5.42 (5.18 to 5.67)	-0.54 (-0.60 to -0.48)	0.007
Absolute change from baseline to 45-days, mmol/L	-1.11 (-1.46 to -0.77) ***	-0.51 (-0.82 to -0.20) **	-0.61 (-0.64 to -0.57)	0.009
Percentage change from baseline to 45-days, %	-18.00 (-23.23 to -12.77)	-7.70 (-12.64 to -2.76)	-10.30 (-10.59 to -10.02)	0.005
120-days, mmol/L	5.12 (4.79 to 5.45) ***	5.64 (5.42 to 5.86) *	-0.52 (-0.63 to -0.41)	0.010
Absolute change from baseline to 120-days, mmol/L	-0.88 (-1.16 to -0.60)	-0.29 (-0.55 to -0.04)	-0.59 (-0.61 to -0.56)	0.003
Percentage change from baseline to 120-days, %	-14.41 (-19.09 to -9.73)	-4.21 (-8.20 to -0.22)	-10.20 (-10.88 to -9.52)	0.001
TG				
Baseline, mmol/L	2.51 (2.20 to 2.82)	2.72 (2.34 to 3.09)	-0.20 (-0.14 to -0.27)	0.355
45-days, mmol/L	1.95 (1.60 to 2.30) **	2.24 (1.92 to 2.55) *	-0.29 (-0.32 to -0.26)	0.138
Absolute change from baseline to 45-days, mmol/L	-0.56 (-0.98 to -0.15)	-0.48 (-0.85 to -0.11)	-0.08 (-0.13 to -0.04)	0.757
Percentage change from baseline to 45-days, %	-16.96 (-37.61 to 3.70)	-12.87 (-26.63 to 0.88)	-4.08 (-10.98 to 2.82)	0.223
120-days, mmol/L	1.96 (1.63 to 2.29) **	2.33 (2.06 to 2.59) **	-0.37 (-0.43 to -0.30)	0.041
Absolute change from baseline to 120-days, mmol/L	-0.55 (-0.97 to -0.13)	-0.39 (-0.68 to -0.10)	-0.16 (-0.29 to -0.03)	0.305
Percentage change from baseline to 120-days, %	-15.67 (-35.99 to 4.64)	-9.90 (-19.77 to -0.03)	-5.78 (-16.22 to 4.67)	0.140
LDL-C				
Baseline, mmol/L	3.71 (3.52 to 3.91)	3.66 (3.45 to 3.87)	0.05 (0.07 to 0.03)	0.720
45-days, mmol/L	2.99 (2.71 to 3.27) ***	3.52 (3.35 to 3.68)	-0.53 (-0.64 to -0.42)	0.002
Absolute change from baseline to 45-days, mmol/L	-0.73 (-0.95 to -0.50)	-0.15 (-0.31 to 0.01)	-0.58 (-0.64 to -0.51)	<0.001
Percentage change from baseline to 45-days, %	-19.57 (-25.52 to -13.63)	-3.15 (-7.53 to 1.23)	-16.43 (-17.99 to -14.87)	<0.001
120-days, mmol/L	3.27 (2.99 to 3.55) ***	3.80 (3.61 to 3.98)	-0.53 (-0.62 to -0.43)	0.003
Absolute change from baseline to 120-days, mmol/L	-0.44 (-0.65 to -0.24)	0.13 (-0.02 to 0.29)	-0.58 (-0.62 to -0.53)	<0.001
Percentage change from baseline to 120-days, %	-12.04 (-17.40 to -6.67)	4.43 (0.12 to 8.73)	-16.46 (-17.52 to -15.41)	<0.001
non-HDL-C				
Baseline, mmol/L	4.54 (4.27 to 4.80)	4.55 (4.28 to 4.83)	-0.02 (-0.01 to -0.02)	0.926
45-days, mmol/L	3.42 (3.08 to 3.76) ***	4.09 (3.86 to 4.32) **	-0.67 (-0.78 to -0.55)	0.002
Absolute change from baseline to 45-days, mmol/L	-1.11 (-1.44 to -0.79)	-0.46 (-0.74 to -0.19)	-0.65 (-0.70 to -0.60)	0.003
Percentage change from baseline to 45-days, %	-24.11 (-31.07 to -17.15)	-8.95 (-14.83 to -3.07)	-15.16 (-16.24 to -14.08)	0.001
120-days, mmol/L	3.59 (3.26 to 3.92) ***	4.18 (3.99 to 4.37) **	-0.59 (-0.73 to -0.46)	0.003
Absolute change from baseline to 120-days, mmol/L	-0.95 (-1.23 to -0.67)	-0.37 (-0.61 to -0.14)	-0.58 (-0.63 to -0.53)	0.002
Percentage change from baseline to 120-days, %	-20.58 (-26.83 to -14.32)	-7.11 (-11.70 to -2.51)	-13.47 (-15.13 to -11.81)	0.001
LDL-C to HDL-C ratio				
Baseline	2.66 (2.37 to 2.94)	2.74 (2.46 to 3.02)	-0.08 (-0.08 to -0.08)	0.751
45-days	2.16 (1.84 to 2.49) ***	2.70 (2.45 to 2.95)	-0.54 (-0.61 to -0.47)	0.002

End outcomes	NMS	Placebo	NMS vs. placebo	
	mean（95%CI)		mean difference (95%CI)	*P* value
Absolute change from baseline to 45-days	-0.49 (-0.62 to -0.37)	-0.03 (-0.11 to 0.04)	-0.46 (-0.51 to -0.41)	<0.001
Percentage change from baseline to 45-days, %	-20.03 (-25.60 to -14.47)	-0.58 (-3.57 to 2.41)	-19.46 (-22.04 to -16.88)	<0.001
120-days	2.24 (1.93 to 2.55) ***	2.66 (2.44 to 2.89)	-0.42 (-0.51 to -0.34)	0.003
Absolute change from baseline to 120-days	-0.42 (-0.56 to -0.27)	-0.07 (-0.20 to 0.05)	-0.34 (-0.37 to -0.32)	0.001
Percentage change from baseline to 120-days, %	-16.14 (-21.41 to -10.88)	-1.48 (-5.20 to 2.23)	-14.66 (-16.21 to -13.11)	<0.001
HDL-C				
Baseline, mmol/L	1.46 (1.33 to 1.60)	1.38 (1.29 to 1.47)	0.09 (0.04 to 0.13)	0.332
45-days, mmol/L	1.46 (1.34 to 1.58)	1.33 (1.26 to 1.41)	0.13 (0.09 to 0.17)	0.167
Absolute change from baseline to 45-days, mmol/L	0.00 (-0.04 to 0.04)	-0.04 (-0.09 to 0.01)	0.04 (0.05 to 0.03)	0.193
Percentage change from baseline to 45-days, %	0.55 (-2.26 to 3.37)	-2.60 (-5.70 to 0.51)	3.15 (3.44 to 2.87)	0.161
120-days, mmol/L	1.53 (1.41 to 1.66) **	1.46 (1.37 to 1.55) *	0.07 (0.04 to 0.10)	0.735
Absolute change from baseline to 120-days, mmol/L	0.07 (0.03 to 0.11)	0.08 (0.02 to 0.14)	-0.01 (0.01 to -0.04)	0.702
Percentage change from baseline to 120-days, %	5.21 (2.36 to 8.05)	6.51 (1.93 to 11.09)	-1.30 (0.43 to -3.04)	0.621
Second outcomes				
Right CIMT				
Baseline, mm	0.84 (0.76 to 0.91)	0.73 (0.66 to 0.80)	0.11 (0.11 to 0.12)	0.023
120-days, mm	0.83 (0.76 to 0.89)	0.77 (0.71 to 0.84)	0.05 (0.06 to 0.05)	0.145
Absolute change from baseline to 120-days, mm	-0.01 (-0.05 to 0.02)	0.05 (-0.02 to 0.11)	-0.06 (-0.03 to -0.09)	0.127
Percentage change from baseline to 120-days, %	-0.22 (-4.40 to 3.95)	9.26 (0.05 to 18.47)	-9.48 (-4.45 to -14.51)	0.144
Left CIMT				
Baseline, mm	0.87 (0.78 to 0.96)	0.80 (0.71 to 0.90)	0.07 (0.07 to 0.06)	0.229
120-days, mm	0.88 (0.79 to 0.97)	0.81 (0.73 to 0.89)	0.07 (0.06 to 0.07)	0.294
Absolute change from baseline to 120-days, mm	0.01 (-0.03 to 0.05)	0.01 (-0.06 to 0.07)	0.00 (0.03 to -0.03)	0.952
Percentage change from baseline to 120-days, %	1.69 (-3.45 to 6.82)	3.86 (-4.83 to 12.54)	-2.17 (1.38 to -5.72)	0.904
Average (Left and right) CIMT				
Baseline, mm	0.85 (0.78 to 0.93)	0.77 (0.69 to 0.84)	0.09 (0.09 to 0.09)	0.084
120-days, mm	0.85 (0.78 to 0.92)	0.79 (0.72 to 0.86)	0.06 (0.06 to 0.06)	0.224
Absolute change from baseline to 120-days, mm	-0.01 (-0.03 to 0.02)	0.03 (-0.02 to 0.08)	-0.03 (-0.01 to -0.06)	0.588
Percentage change from baseline to 120-days, %	-0.04 (-3.06 to 2.98)	5.17 (-2.09 to 12.42)	-5.21 (-0.98 to -9.44)	0.583
Over 60 years	NMS (*n*=29)	Placebo (*n*=32)		
Primary outcomes				
TC				
Baseline, mmol/L	5.97 (5.64 to 6.31)	5.80 (5.41 to 6.18)	0.17 (0.22 to 0.13)	0.398
45-days, mmol/L	5.22 (4.97 to 5.48) ***	5.61 (5.36 to 5.86)	-0.39 (-0.39 to -0.38)	0.031
Absolute change from baseline to 45-days, mmol/L	-0.75 (-1.03 to -0.47)	-0.19 (-0.45 to 0.08)	-0.56 (-0.58 to -0.54)	0.004
Percentage change from baseline to 45-days, %	-11.83 (-15.94 to -7.73)	-1.71 (-6.06 to 2.64)	-10.12 (-9.88 to -10.36)	0.001
120-days, mmol/L	5.17 (4.84 to 5.50) ***	5.46 (5.21 to 5.72) *	-0.29 (-0.37 to -0.22)	0.155
Absolute change from baseline to 120-days, mmol/L	-0.80 (-1.08 to -0.53)	-0.34 (-0.65 to -0.03)	-0.47 (-0.43 to -0.51)	0.024
Percentage change from baseline to 120-days, %	-13.15 (-17.44 to -8.87)	-4.12 (-8.94 to 0.69)	-9.03 (-8.50 to -9.56)	0.006
TG				
Baseline, mmol/L	2.79 (2.27 to 3.31)	2.76 (2.37 to 3.15)	0.03 (-0.09 to 0.16)	0.756
45-days, mmol/L	2.20 (1.69 to 2.71) **	2.46 (2.09 to 2.82) *	-0.25 (-0.40 to -0.11)	0.101
Absolute change from baseline to 45-days, mmol/L	-0.59 (-0.96 to -0.22)	-0.30 (-0.67 to 0.07)	-0.29 (-0.29 to -0.29)	0.285
Percentage change from baseline to 45-days, %	-18.10 (-29.54 to -6.66)	-5.64 (-17.35 to 6.07)	-12.46 (-12.19 to -12.73)	0.133

End outcomes	NMS	Placebo	NMS vs. placebo	
	mean（95%CI)		mean difference (95%CI)	*P* value
120-days, mmol/L	2.16 (1.81 to 2.51) ***	2.44 (2.05 to 2.83) *	-0.28 (-0.24 to -0.32)	0.402
Absolute change from baseline to 120-days, mmol/L	-0.63 (-0.98 to -0.28)	-0.32 (-0.67 to 0.04)	-0.31 (-0.30 to -0.32)	0.279
Percentage change from baseline to 120-days, %	-16.48 (-28.43 to -4.54)	-5.52 (-20.02 to 8.97)	-10.96 (-8.41 to -13.51)	0.245
LDL-C				
Baseline, mmol/L	3.62 (3.33 to 3.91)	3.48 (3.24 to 3.72)	0.14 (0.09 to 0.19)	0.691
45-days, mmol/L	3.17 (2.96 to 3.39) ***	3.47 (3.28 to 3.65)	-0.29 (-0.32 to -0.27)	0.003
Absolute change from baseline to 45-days, mmol/L	-0.45 (-0.63 to -0.26)	-0.01 (-0.16 to 0.14)	-0.43 (-0.46 to -0.40)	<0.001
Percentage change from baseline to 45-days, %	-11.27 (-15.66 to -6.88)	1.19 (-3.33 to 5.70)	-12.46 (-12.33 to -12.58)	<0.001
120-days, mmol/L	3.24 (2.95 to 3.54) ***	3.42 (3.20 to 3.63)	-0.17 (-0.26 to -0.08)	0.124
Absolute change from baseline to 120-days, mmol/L	-0.37 (-0.57 to -0.18)	-0.06 (-0.24 to 0.11)	-0.31 (-0.33 to -0.29)	0.019
Percentage change from baseline to 120-days, %	-10.04 (-15.54 to -4.55)	-0.28 (-5.44 to 4.88)	-9.76 (-10.10 to -9.43)	0.010
non-HDL-C				
Baseline, mmol/L	4.54 (4.20 to 4.89)	4.39 (4.05 to 4.72)	0.15 (0.15 to 0.16)	0.723
45-days, mmol/L	3.72 (3.45 to 3.98) ***	4.14 (3.92 to 4.36) *	-0.42 (-0.47 to -0.38)	0.015
Absolute change from baseline to 45-days, mmol/L	-0.82 (-1.10 to -0.55)	-0.25 (-0.49 to -0.01)	-0.58 (-0.61 to -0.54)	0.002
Percentage change from baseline to 45-days, %	-17.24 (-22.48 to -12.00)	-3.71 (-8.69 to 1.27)	-13.53 (-13.79 to -13.27)	<0.001
120-days, mmol/L	3.62 (3.29 to 3.95) ***	3.94 (3.71 to 4.17) **	-0.32 (-0.42 to -0.22)	0.108
Absolute change from baseline to 120-days, mmol/L	-0.92 (-1.20 to -0.64)	-0.44 (-0.75 to -0.13)	-0.48 (-0.44 to -0.51)	0.023
Percentage change from baseline to 120-days, %	-19.82 (-25.53 to -14.11)	-7.74 (-13.82 to -1.65)	-12.09 (-11.71 to -12.46)	0.004
LDL-C to HDL-C ratio				
Baseline	2.61 (2.35 to 2.88)	2.51 (2.34 to 2.68)	0.11 (0.02 to 0.20)	0.736
45-days	2.18 (1.98 to 2.38) ***	2.41 (2.24 to 2.58) *	-0.23 (-0.27 to -0.20)	0.077
Absolute change from baseline to 45-days	-0.44 (-0.57 to -0.30)	-0.10 (-0.18 to -0.01)	-0.34 (-0.38 to -0.29)	<0.001
Percentage change from baseline to 45-days, %	-15.68 (-20.08 to -11.28)	-3.21 (-6.82 to 0.40)	-12.47 (-13.26 to -11.68)	<0.001
120-days	2.17 (1.94 to 2.40) ***	2.31 (2.14 to 2.49) *	-0.15 (-0.20 to -0.09)	0.301
Absolute change from baseline to 120-days	-0.45 (-0.59 to -0.30)	-0.20 (-0.33 to -0.06)	-0.25 (-0.27 to -0.24)	0.011
Percentage change from baseline to 120-days, %	-16.46 (-22.18 to -10.74)	-6.65 (-11.88 to -1.42)	-9.81 (-10.30 to -9.32)	0.012
HDL-C				
Baseline, mmol/L	1.43 (1.33 to 1.54)	1.41 (1.32 to 1.51)	0.02 (0.01 to 0.03)	0.823
45-days, mmol/L	1.51 (1.40 to 1.62) **	1.47 (1.38 to 1.57)	0.04 (0.02 to 0.05)	0.778
Absolute change from baseline to 45-days, mmol/L	0.08 (0.02 to 0.13)	0.06 (0.00 to 0.12)	0.02 (0.02 to 0.01)	0.397
Percentage change from baseline to 45-days, %	5.72 (1.99 to 9.45)	5.07 (0.45 to 9.69)	0.65 (1.54 to -0.24)	0.346
120-days, mmol/L	1.55 (1.44 to 1.65) ***	1.52 (1.40 to 1.63) **	0.03 (0.03 to 0.02)	0.525
Absolute change from baseline to 120-days, mmol/L	0.11 (0.05 to 0.18)	0.11 (0.04 to 0.17)	0.01 (0.01 to 0.00)	0.880
Percentage change from baseline to 120-days, %	8.66 (4.06 to 13.26)	7.92 (3.13 to 12.72)	0.74 (0.93 to 0.55)	0.456
Second outcomes				
Right CIMT				
Baseline, mm	0.83 (0.75 to 0.91)	0.88 (0.81 to 0.95)	-0.05 (-0.07 to -0.04)	0.397
120-days, mm	0.87 (0.80 to 0.94)	0.93 (0.87 to 0.99)	-0.06 (-0.07 to -0.06)	0.171
Absolute change from baseline to 120-days, mm	0.04 (-0.05 to 0.13)	0.05 (0.00 to 0.10)	-0.01 (-0.05 to 0.03)	0.907
Percentage change from baseline to 120-days, %	12.49 (-3.64 to 28.62)	7.78 (0.57 to 14.99)	4.71 (-4.21 to 13.63)	0.907
Left CIMT				
Baseline, mm	0.88 (0.81 to 0.96)	0.94 (0.87 to 1.02)	-0.06 (-0.06 to -0.06)	0.237

End outcomes	NMS	Placebo	NMS vs. placebo	
	mean（95%CI）		mean difference (95%CI)	*P* value
120-days, mm	0.90 (0.83 to 0.97)	0.94 (0.87 to 1.02)	-0.04 (-0.04 to -0.04)	0.369
Absolute change from baseline to 120-days, mm	0.02 (-0.03 to 0.07)	0.00 (-0.07 to 0.07)	0.02 (0.04 to 0.00)	0.846
Percentage change from baseline to 120-days, %	3.74 (-3.46 to 10.95)	2.41 (-5.32 to 10.14)	1.34 (1.87 to 0.81)	0.871
Average (Left and right) CIMT				
Baseline, mm	0.86 (0.79 to 0.93)	0.91 (0.85 to 0.97)	-0.06 (-0.06 to -0.05)	0.228
120-days, mm	0.88 (0.82 to 0.95)	0.94 (0.88 to 1.00)	-0.05 (-0.06 to -0.05)	0.205
Absolute change from baseline to 120-days, mm	0.03 (-0.04 to 0.09)	0.03 (-0.03 to 0.08)	0.00 (-0.01 to 0.01)	0.773
Percentage change from baseline to 120-days, %	5.73 (-3.07 to 14.53)	4.50 (-1.81 to 10.81)	1.24 (-1.26 to 3.73)	0.785

Mean difference refers to the disparity between two groups. The difference is considered statistically significant when P<0.05, denoted as *; when P<0.01, denoted as **; and when P<0.001, denoted as ***.

Subgroup analyses by sex were generally consistent with the previous results (Supplement Table S8).

Supplement Table S8 Primary and second outcomes under sex subgroups

End outcomes	NMS	Placebo	NMS vs. placebo	
	mean（95%CI）		mean difference (95%CI)	*P* value
Female	NMS (*n*=45)	Placebo (*n*=41)		
Primary outcomes				
TC				
Baseline, mmol/L	5.93 (5.65 to 6.22)	6.00 (5.75 to 6.26)	-0.07 (-0.10 to -0.04)	0.709
45-days, mmol/L	5.56 (5.36 to 5.77) ***	5.14 (4.91 to 5.37) **	0.42 (0.44 to 0.40)	0.007
Absolute change from baseline to 45-days, mmol/L	-0.37 (-0.60 to -0.14)	-0.86 (-1.09 to -0.63)	0.49 (0.49 to 0.49)	0.003
Percentage change from baseline to 45-days, %	-4.97 (-8.65 to -1.30)	-13.79 (-17.30 to -10.28)	8.82 (8.65 to 8.98)	<0.001
120-days, mmol/L	5.55 (5.35 to 5.75) ***	5.18 (4.92 to 5.44) **	0.37 (0.43 to 0.32)	0.024
Absolute change from baseline to 120-days, mmol/L	-0.38 (-0.63 to -0.13)	-0.83 (-1.05 to -0.60)	0.44 (0.42 to 0.46)	0.010
Percentage change from baseline to 120-days, %	-5.09 (-8.90 to -1.28)	-13.35 (-16.94 to -9.76)	8.26 (8.04 to 8.47)	0.002
TG				
Baseline, mmol/L	2.77 (2.46 to 3.08)	2.64 (2.25 to 3.02)	0.13 (0.21 to 0.06)	0.254
45-days, mmol/L	2.43 (2.15 to 2.72) ***	2.12 (1.72 to 2.51) *	0.32 (0.42 to 0.21)	0.020
Absolute change from baseline to 45-days, mmol/L	-0.33 (-0.63 to -0.04)	-0.52 (-0.84 to -0.19)	0.18 (0.21 to 0.16)	0.303
Percentage change from baseline to 45-days, %	-7.60 (-17.32 to 2.12)	-15.48 (-29.16 to -1.80)	7.88 (11.84 to 3.92)	0.098
120-days, mmol/L	2.34 (2.04 to 2.63) ***	2.05 (1.76 to 2.33) ***	0.29 (0.29 to 0.30)	0.116
Absolute change from baseline to 120-days, mmol/L	-0.43 (-0.70 to -0.16)	-0.59 (-0.90 to -0.27)	0.16 (0.20 to 0.11)	0.497
Percentage change from baseline to 120-days, %	-11.94 (-20.55 to -3.33)	-15.41 (-29.08 to -1.75)	3.48 (8.53 to -1.58)	0.287
LDL-C				
Baseline, mmol/L	3.53 (3.35 to 3.72)	3.68 (3.46 to 3.89)	-0.14 (-0.12 to -0.16)	0.528
45-days, mmol/L	3.45 (3.31 to 3.59) ***	3.13 (2.93 to 3.32)	0.32 (0.37 to 0.27)	<0.001
Absolute change from baseline to 45-days, mmol/L	-0.08 (-0.21 to 0.04)	-0.55 (-0.71 to -0.39)	0.46 (0.50 to 0.43)	<0.001
Percentage change from baseline to 45-days, %	-1.01 (-4.61 to 2.59)	-14.25 (-18.30 to -10.21)	13.25 (13.69 to 12.80)	<0.001

End outcomes	NMS	Placebo	NMS vs. placebo	
	mean（95%CI)		mean difference (95%CI)	*P* value
120-days, mmol/L	3.52 (3.35 to 3.68) ***	3.25 (3.02 to 3.48)	0.27 (0.33 to 0.20)	0.010
Absolute change from baseline to 120-days, mmol/L	-0.02 (-0.17 to 0.13)	-0.43 (-0.60 to -0.25)	0.41 (0.43 to 0.38)	<0.001
Percentage change from baseline to 120-days, %	0.83 (-3.37 to 5.02)	-11.18 (-15.84 to -6.52)	12.01 (12.47 to 11.54)	<0.001
non-HDL-C				
Baseline, mmol/L	4.48 (4.23 to 4.74)	4.54 (4.28 to 4.80)	-0.05 (-0.05 to -0.06)	0.955
45-days, mmol/L	4.11 (3.92 to 4.29) ***	3.63 (3.40 to 3.87) ***	0.47 (0.53 to 0.42)	<0.001
Absolute change from baseline to 45-days, mmol/L	-0.38 (-0.58 to -0.17)	-0.91 (-1.13 to -0.68)	0.53 (0.55 to 0.51)	<0.001
Percentage change from baseline to 45-days, %	-6.78 (-11.03 to -2.54)	-19.30 (-23.77 to -14.83)	12.52 (12.74 to 12.29)	<0.001
120-days, mmol/L	4.00 (3.82 to 4.18) ***	3.60 (3.34 to 3.86) ***	0.40 (0.48 to 0.31)	0.003
Absolute change from baseline to 120-days, mmol/L	-0.49 (-0.73 to -0.25)	-0.94 (-1.17 to -0.70)	0.45 (0.45 to 0.46)	0.004
Percentage change from baseline to 120-days, %	-8.95 (-13.56 to -4.34)	-20.15 (-25.00 to -15.30)	11.20 (11.44 to 10.96)	<0.001
LDL-C to HDL-C ratio				
Baseline	2.48 (2.34 to 2.61)	2.59 (2.38 to 2.80)	-0.12 (-0.04 to -0.19)	0.600
45-days	2.41 (2.28 to 2.53) ***	2.15 (1.95 to 2.35)	0.26 (0.34 to 0.19)	0.005
Absolute change from baseline to 45-days	-0.07 (-0.14 to -0.00)	-0.45 (-0.55 to -0.34)	0.38 (0.42 to 0.34)	<0.001
Percentage change from baseline to 45-days, %	-2.00 (-4.89 to 0.89)	-16.97 (-20.81 to -13.12)	14.97 (15.93 to 14.01)	<0.001
120-days	2.31 (2.18 to 2.43) ***	2.13 (1.92 to 2.33) **	0.18 (0.26 to 0.10)	0.027
Absolute change from baseline to 120-days	-0.17 (-0.27 to -0.07)	-0.46 (-0.59 to -0.34)	0.29 (0.32 to 0.26)	<0.001
Percentage change from baseline to 120-days, %	-5.77 (-9.63 to -1.92)	-17.44 (-22.30 to -12.58)	11.67 (12.68 to 10.66)	<0.001
HDL-C				
Baseline, mmol/L	1.45 (1.38 to 1.52)	1.47 (1.38 to 1.55)	-0.02 (-0.00 to -0.03)	0.938
45-days, mmol/L	1.46 (1.39 to 1.53) *	1.51 (1.42 to 1.60)	-0.05 (-0.03 to -0.07)	0.460
Absolute change from baseline to 45-days, mmol/L	0.01 (-0.04 to 0.06)	0.05 (0.01 to 0.08)	-0.04 (-0.05 to -0.03)	0.224
Percentage change from baseline to 45-days, %	1.44 (-2.09 to 4.98)	3.51 (0.83 to 6.20)	-2.07 (-2.92 to -1.22)	0.197
120-days, mmol/L	1.55 (1.47 to 1.64) ***	1.58 (1.49 to 1.67) ***	-0.03 (-0.02 to -0.03)	0.616
Absolute change from baseline to 120-days, mmol/L	0.10 (0.05 to 0.16)	0.11 (0.07 to 0.16)	-0.01 (-0.02 to 0.00)	0.382
Percentage change from baseline to 120-days, %	7.84 (3.93 to 11.74)	8.41 (5.19 to 11.63)	-0.57 (-1.26 to 0.11)	0.416
Second outcomes				
Right CIMT				
Baseline, mm	0.81 (0.75 to 0.87)	0.81 (0.75 to 0.87)	-0.00 (0.00 to -0.01)	0.930
120-days, mm	0.86 (0.81 to 0.92)	0.84 (0.79 to 0.89) *	0.03 (0.02 to 0.03)	0.499
Absolute change from baseline to 120-days, mm	0.06 (0.01 to 0.10)	0.03 (-0.03 to 0.09)	0.03 (0.04 to 0.01)	0.363
Percentage change from baseline to 120-days, %	9.53 (2.87 to 16.18)	8.55 (-2.38 to 19.48)	0.98 (5.25 to -3.30)	0.369
Left CIMT				
Baseline, mm	0.87 (0.80 to 0.93)	0.85 (0.79 to 0.91)	0.02 (0.02 to 0.02)	0.683
120-days, mm	0.88 (0.82 to 0.93)	0.87 (0.81 to 0.93)	0.01 (0.01 to 0.01)	0.818
Absolute change from baseline to 120-days, mm	0.01 (-0.04 to 0.07)	0.02 (-0.02 to 0.06)	-0.01 (-0.03 to 0.01)	0.798
Percentage change from baseline to 120-days, %	4.19 (-2.67 to 11.05)	3.82 (-1.48 to 9.12)	0.37 (-1.19 to 1.93)	0.932
Average (Left and right) CIMT				
Baseline, mm	0.84 (0.79 to 0.89)	0.83 (0.77 to 0.89)	0.01 (0.01 to 0.00)	0.886
120-days, mm	0.87 (0.82 to 0.92)	0.85 (0.80 to 0.90)	0.02 (0.02 to 0.02)	0.581
Absolute change from baseline to 120-days, mm	0.03 (-0.01 to 0.08)	0.02 (-0.02 to 0.06)	0.01 (0.01 to 0.01)	0.665
Percentage change from baseline to 120-days, %	5.88 (0.24 to 11.52)	4.23 (-1.67 to 10.12)	1.65 (1.91 to 1.40)	0.682
Male	NMS (*n*=13)	Placebo (*n*=14)		

End outcomes	NMS	Placebo	NMS vs. placebo	
	mean（95%CI)		mean difference (95%CI)	*P* value
Primary outcomes				
TC				
Baseline, mmol/L	5.60 (5.15 to 6.05)	5.93 (5.49 to 6.37)	-0.33 (-0.34 to -0.32)	0.356
45-days, mmol/L	5.40 (5.07 to 5.72) ***	4.83 (4.41 to 5.24)	0.57 (0.67 to 0.47)	0.024
Absolute change from baseline to 45-days, mmol/L	-0.20 (-0.65 to 0.25)	-1.10 (-1.69 to -0.51)	0.90 (1.04 to 0.76)	0.024
Percentage change from baseline to 45-days, %	-2.40 (-10.42 to 5.63)	-17.56 (-26.17 to -8.94)	15.16 (15.75 to 14.57)	0.019
120-days, mmol/L	5.51 (5.16 to 5.86) ***	5.04 (4.51 to 5.58)	0.47 (0.65 to 0.28)	0.152
Absolute change from baseline to 120-days, mmol/L	-0.09 (-0.35 to 0.17)	-0.88 (-1.27 to -0.50)	0.80 (0.92 to 0.67)	0.003
Percentage change from baseline to 120-days, %	-0.96 (-5.69 to 3.78)	-14.92 (-21.56 to -8.28)	13.96 (15.87 to 12.05)	0.002
TG				
Baseline, mmol/L	2.64 (2.07 to 3.20)	2.73 (2.32 to 3.14)	-0.09 (-0.24 to 0.06)	0.627
45-days, mmol/L	2.10 (1.66 to 2.54) *	1.98 (1.56 to 2.39)	0.12 (0.09 to 0.15)	0.593
Absolute change from baseline to 45-days, mmol/L	-0.54 (-1.11 to 0.03)	-0.75 (-1.27 to -0.24)	0.21 (0.16 to 0.26)	0.519
Percentage change from baseline to 45-days, %	-13.33 (-35.42 to 8.77)	-23.65 (-43.19 to -4.12)	10.33 (7.77 to 12.89)	0.430
120-days, mmol/L	2.57 (2.19 to 2.95) *	2.12 (1.68 to 2.57)	0.44 (0.50 to 0.39)	0.094
Absolute change from baseline to 120-days, mmol/L	-0.07 (-0.52 to 0.38)	-0.61 (-1.13 to -0.08)	0.54 (0.61 to 0.47)	0.062
Percentage change from baseline to 120-days, %	7.93 (-19.75 to 35.61)	-18.11 (-38.23 to 2.01)	26.04 (18.47 to 33.60)	0.094
LDL-C				
Baseline, mmol/L	3.65 (3.30 to 4.00)	3.63 (3.30 to 3.96)	0.03 (0.01 to 0.04)	0.903
45-days, mmol/L	3.62 (3.34 to 3.90) ***	2.96 (2.57 to 3.36)	0.65 (0.77 to 0.54)	0.009
Absolute change from baseline to 45-days, mmol/L	-0.03 (-0.29 to 0.22)	-0.66 (-1.02 to -0.31)	0.63 (0.73 to 0.52)	0.006
Percentage change from baseline to 45-days, %	0.12 (-7.17 to 7.40)	-17.95 (-27.25 to -8.66)	18.07 (20.08 to 16.06)	0.005
120-days, mmol/L	3.83 (3.51 to 4.15) **	3.28 (2.83 to 3.72) *	0.55 (0.68 to 0.42)	0.037
Absolute change from baseline to 120-days, mmol/L	0.17 (0.01 to 0.34)	-0.35 (-0.56 to -0.14)	0.52 (0.57 to 0.48)	0.001
Percentage change from baseline to 120-days, %	5.31 (0.61 to 10.01)	-10.42 (-16.79 to -4.04)	15.72 (17.40 to 14.05)	0.001
non-HDL-C				
Baseline, mmol/L	4.38 (3.94 to 4.82)	4.54 (4.14 to 4.93)	-0.16 (-0.20 to -0.11)	0.627
45-days, mmol/L	4.15 (3.83 to 4.47) ***	3.41 (2.91 to 3.91)	0.74 (0.92 to 0.55)	0.019
Absolute change from baseline to 45-days, mmol/L	-0.23 (-0.63 to 0.17)	-1.12 (-1.67 to -0.57)	0.89 (1.05 to 0.74)	0.011
Percentage change from baseline to 45-days, %	-3.56 (-12.64 to 5.53)	-23.96 (-35.42 to -12.50)	20.40 (22.79 to 18.02)	0.006
120-days, mmol/L	4.23 (3.92 to 4.54) ***	3.62 (3.12 to 4.12)	0.60 (0.80 to 0.41)	0.052
Absolute change from baseline to 120-days, mmol/L	-0.15 (-0.43 to 0.13)	-0.91 (-1.28 to -0.55)	0.76 (0.85 to 0.67)	0.003
Percentage change from baseline to 120-days, %	-2.27 (-8.44 to 3.90)	-20.26 (-28.84 to -11.67)	17.99 (20.40 to 15.57)	0.002
LDL-C to HDL-C ratio				
Baseline	3.07 (2.60 to 3.54)	2.75 (2.32 to 3.19)	0.32 (0.29 to 0.35)	0.290
45-days	3.00 (2.55 to 3.44) ***	2.24 (1.79 to 2.69)	0.76 (0.76 to 0.75)	0.015
Absolute change from baseline to 45-days	-0.07 (-0.19 to 0.05)	-0.51 (-0.71 to -0.31)	0.44 (0.51 to 0.36)	<0.001
Percentage change from baseline to 45-days, %	-2.14 (-6.13 to 1.85)	-20.00 (-28.36 to -11.64)	17.86 (22.23 to 13.49)	<0.001
120-days	3.04 (2.70 to 3.38) ***	2.41 (1.98 to 2.84)	0.63 (0.72 to 0.54)	0.020
Absolute change from baseline to 120-days	-0.03 (-0.26 to 0.20)	-0.34 (-0.48 to -0.20)	0.31 (0.22 to 0.40)	0.021
Percentage change from baseline to 120-days, %	0.64 (-5.80 to 7.08)	-13.00 (-17.73 to -8.26)	13.64 (11.93 to 15.34)	0.001
HDL-C				

End outcomes	NMS	Placebo	NMS vs. placebo	
	mean（95%CI)		mean difference (95%CI)	*P* value
Baseline, mmol/L	1.22 (1.11 to 1.32)	1.39 (1.18 to 1.60)	-0.17 (-0.07 to -0.28)	0.126
45-days, mmol/L	1.24 (1.12 to 1.36)	1.41 (1.23 to 1.59)	-0.17 (-0.11 to -0.22)	0.106
Absolute change from baseline to 45-days, mmol/L	0.03 (-0.05 to 0.10)	0.02 (-0.06 to 0.10)	0.01 (0.01 to 0.00)	0.906
Percentage change from baseline to 45-days, %	2.29 (-4.00 to 8.57)	2.58 (-3.33 to 8.50)	-0.30 (-0.67 to 0.08)	0.941
120-days, mmol/L	1.28 (1.16 to 1.40)	1.42 (1.23 to 1.61)	-0.14 (-0.07 to -0.20)	0.198
Absolute change from baseline to 120-days, mmol/L	0.06 (-0.02 to 0.14)	0.03 (-0.04 to 0.09)	0.03 (0.02 to 0.05)	0.474
Percentage change from baseline to 120-days, %	5.39 (-0.70 to 11.48)	2.98 (-2.15 to 8.12)	2.41 (1.45 to 3.37)	0.518
Second outcomes				
Right CIMT				
Baseline, mm	0.82 (0.68 to 0.96)	0.90 (0.78 to 1.02)	-0.08 (-0.10 to -0.05)	0.375
120-days, mm	0.85 (0.71 to 0.98)	0.88 (0.77 to 0.99)	-0.03 (-0.06 to -0.01)	0.605
Absolute change from baseline to 120-days, mm	0.02 (-0.07 to 0.11)	-0.02 (-0.13 to 0.09)	0.04 (0.07 to 0.02)	0.507
Percentage change from baseline to 120-days, %	4.69 (-5.78 to 15.16)	0.42 (-12.57 to 13.41)	4.27 (6.79 to 1.75)	0.542
Left CIMT				
Baseline, mm	0.93 (0.75 to 1.11)	0.96 (0.83 to 1.09)	-0.03 (-0.08 to 0.02)	0.770
120-days, mm	0.91 (0.74 to 1.07)	0.95 (0.82 to 1.08)	-0.04 (-0.07 to -0.01)	0.695
Absolute change from baseline to 120-days, mm	-0.02 (-0.13 to 0.08)	-0.01 (-0.07 to 0.06)	-0.02 (-0.06 to 0.02)	0.695
Percentage change from baseline to 120-days, %	-0.87 (-9.85 to 8.12)	-0.30 (-8.59 to 7.99)	-0.57 (-1.27 to 0.13)	0.674
Average (Left and right) CIMT				
Baseline, mm	0.88 (0.73 to 1.03)	0.93 (0.81 to 1.04)	-0.05 (-0.09 to -0.02)	0.626
120-days, mm	0.88 (0.73 to 1.02)	0.91 (0.80 to 1.02)	-0.04 (-0.07 to -0.00)	0.715
Absolute change from baseline to 120-days, mm	0.00 (-0.07 to 0.07)	-0.01 (-0.08 to 0.05)	0.01 (0.01 to 0.02)	0.512
Percentage change from baseline to 120-days, %	1.04 (-6.39 to 8.48)	-0.59 (-8.83 to 7.65)	1.63 (2.43 to 0.83)	0.464

Mean difference refers to the disparity between two groups. The difference is considered statistically significant when $P<0.05$, denoted as *; when $P<0.01$, denoted as **; and when $P<0.001$, denoted as ***.
